# Genetic counselors' and community clinicians' implementation and perceived barriers to informed consent during pre‐test counseling for hereditary cancer risk

**DOI:** 10.1002/jgc4.1887

**Published:** 2024-03-13

**Authors:** Alexandra Capasso, Bita Nehoray, Nicholas Gorman, Emily A. Quinn, Daiana Bucio, Kathleen R. Blazer

**Affiliations:** ^1^ School of Pharmacy and Health Sciences Keck Graduate Institute Claremont California USA; ^2^ Division of Clinical Cancer Genomics City of Hope National Medical Center Duarte California USA; ^3^ Independent Scholar

**Keywords:** education, genetic counseling, genetics services, risk assessment, service delivery models

## Abstract

As demand for genetic cancer risk assessment (GCRA) continues to increase, so does the sense of urgency to scale up efforts to triage patients, facilitate informed consent, and order genetic testing for cancer risk. The National Society of Genetic Counselors outlines the elements of informed consent that should be addressed in a GCRA session. While this practice resource aims to improve health equity, research on how well the elements of informed consent are implemented in practice is lacking. This retrospective and prospective mixed‐methods study assessed how adequately the elements of informed consent are addressed during pre‐test GCRA among 307 community clinicians (CC) and 129 cancer genetic counselors (GC), and barriers they face to addressing these elements. Results revealed that more than 90% of both cohorts consistently addressed components of at least 5 of the 10 elements of informed consent during a pre‐test consultation. Technical aspects and accuracy of the test and utilization of test results were the most similarly addressed elements. Notably, GCs more often review the purpose of the test and who to test, general information about the gene(s), and economic considerations whereas CCs more often review alternatives to testing. Both cohorts reported psychosocial aspects of the informed consent process as the least adequately addressed element. Time constraints and patient‐related concerns were most often cited by both cohorts as barriers to optimal facilitation of informed consent. Additional barriers reported by CCs included provider lack of awareness, experience, or education, and availability of resources and institutional support. Findings from this study may contribute to the development of alternative delivery models that incorporate supplementary educational tools to enhance patient understanding about the utility of genetic testing, while helping to mitigate the barrier of time constraints. Equally important is the use of this information to develop continuing education tools for providers.

**What is known about this topic**

The NSGC outlines the elements of informed consent that should be addressed prior to testing in a genetic cancer risk assessment session. In response to the ever‐increasing demand for genetic cancer risk assessment services, many providers without formal genetics training in diverse practice settings are involved in facilitating informed consent for hereditary cancer predisposition testing, and several alternate models for streamlining the delivery of these services have emerged.

**What this paper adds to the topic**

This paper assesses which elements of the informed consent process clinicians address during a GCRA session and barriers they face to addressing these elements. This information may inform the development of patient and provider education tools and resources to assist in facilitating the informed consent process.

## 1 INTRODUCTION

Over recent decades more than 150 genes associated with hereditary cancer predisposition have been identified, and the advancement of high‐throughput genomic technologies continues to drive the discovery of both rare and common genetic variants that confer varying degrees of cancer risk (Alvarado et al., 2020: Couch et al., 2017; Graffeo et al., 2022; Kamps et al., 2017; Samadder et al., 2021; Susswein et al., 2016; Tsaousis et al., 2019). Identifying these variants allows for personalized prevention, risk management, early detection, and targeted cancer therapies that have been proven to decrease mortality (de Jong et al., 2006; Domchek et al., 2010).

Genetic cancer risk assessment (GCRA) is an interdisciplinary standard‐of‐care practice that employs genetic and genomic tools to identify individuals and families with inherited cancer risk (Berliner et al., 2021; Weitzel et al., 2011). Rapid and ongoing advances in genetic and genomic technologies, public awareness, reduced cost, and increased accessibility and uptake of both germline and somatic genetic testing continue to accelerate the demand for GCRA services across the spectrum of oncology care (National Human Genome Research Institute, 2021; Tiner et al., 2022; Trivedi et al., 2019; Yap et al., 2021).

These increased demands, paired with a shortage of genetic counselors (GCs) and medical geneticists that is predicted to continue for at least another decade, have prompted a growing number of healthcare providers without formal genetics training to deliver GCRA, particularly in community settings (Hoskovec et al., 2018; Jenkins et al., 2021; Maiese et al., 2019; Radford et al., 2014; Weitzel et al., 2011). Moreover, the demand for testing has led to the development of a number of alternative GCRA delivery models (Cohen et al., 2019; Cohen & Nixon, 2023; Greenberg et al., 2020; McCuaig et al., 2018; Rayes et al., 2019). Some of these models reduce the time needed for pre‐test consultation through the utilization of supplementary tools, and several engage community clinicians to conduct elements of the GCRA process (Giri et al., 2022; Pierle & Mahon, 2019; Trepanier & Allain, 2014). For the purposes of this study, community clinicians are defined as medical providers who deliver GCRA services but do not have advanced degrees in genetics or genetic counseling.

A central goal of GCRA is to ensure that patients are provided with the information and guidance needed to make an informed decision about whether to pursue genetic testing. In its practice resource, the National Society of Genetic Counselors (NSGC) outlines ten essential elements of informed consent for genetic testing in cancer genomics (Riley et al., 2012): (1) The purpose of the test and whom to test; (2) General information about the gene(s); (3) Possible test results; (4) Technical aspects and accuracy of the test; (5) Economic considerations; (6) The possibility of genetic information discrimination; (7) Psychosocial aspects; (8) Confidentiality; (9) Utilization of test results; (10) Alternatives to genetic testing.

While the NSGC practice resource aims to improve health equity, research on how the elements of informed consent are implemented into GCRA practice is lacking. The purpose of this study was to explore the perceptions, experiences, and barriers related to pre‐test counseling for informed consent among GCs and community clinicians who provide GCRA services.

## 2 METHODS

### 2.1 Study design

**FIGURE 1:**
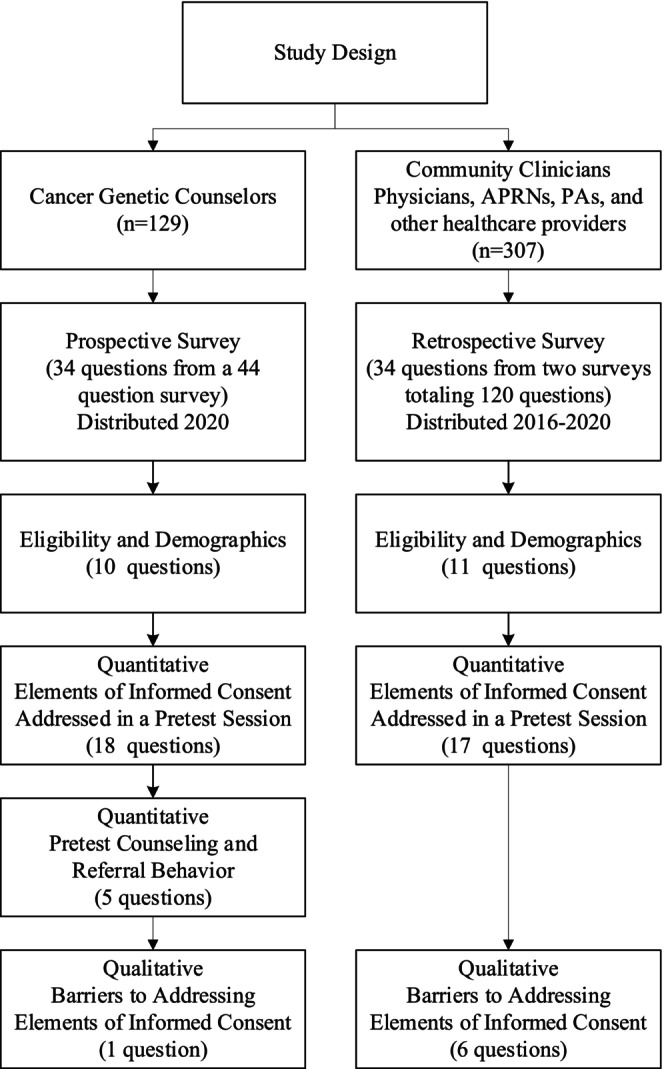
Flowchart of study design.

The study combined a retrospective review and a prospective mixed‐methods design comprised of two participant populations: community clinicians and cancer GCs (Figure 1). The study was classified as exempt by the Claremont Graduate University Institutional Review Board (IRB # 3838).

### 2.2 Participants

Retrospective data were collected from a diverse population of community clinicians during their participation in a comprehensive course in GCRA to support delivery of GCRA services in oncology and cancer screening practices across the United States of America (USA) and internationally. Prospective data were obtained from cancer GCs across the USA and internationally.

#### 2.2.1 Community clinician cohort

The community clinician sample pool was comprised of community‐based clinicians (physicians, advanced practice registered nurses (APRN), physician assistants (PA), GCs, and other healthcare providers) in the USA and internationally, who were participating in a 13‐week National Cancer Institute (NCI) funded interdisciplinary intensive course in clinical cancer genetics between the years 2016 to 2020 and are ongoing members of the program's *Clinical Cancer Genomics Community of Practice* (CCGCoP; Blazer et al., 2004, 2005, 2011, 2012). These community clinicians were actively practicing GCRA and facilitating the informed consent process while enrolled in the course at the time of survey distribution.

#### 2.2.2 Cancer genetic counselor cohort

The GC sample pool was comprised of cancer GCs across the USA and internationally, who were actively practicing GCRA and facilitating the informed consent process from September 2020 to December 2020.

### 2.3 Instrumentation

#### 2.3.1 Community clinician retrospective surveys

Course Application—Demographic data about the community clinician participants (Table 1) was extracted from 11 items of a 35‐item closed and open response application survey completed prior to participating in course. The application took approximately 30 min to complete.

**TABLE 1:** Demographics of participants (*n* = 129–421).

Demographic	Prospective study		Retrospective review				*p*
			Community clinicians^a^				
	Cancer genetic counselor (*n* = 129)		Physician (*n* = 126)		Advanced practice registered nurse or physician assistant (*n* = 166)		
	M (SD)	Freq (%)	M (SD)	Freq (%)	M (SD)	Freq (%)	
Age	30.1 (5.9)		^b^		^b^		
Sex							
Male		4 (3.1)		33 (26.2)		2 (1.2)	<0.001
Female		125 (96.9)		93 (73.8)		164 (98.8)	
Ethnicity							
Hispanic, Latino, or of Spanish origin		5 (3.9)		29 (24.0)		11 (6.8)	<0.001
Non‐Hispanic, Latino, or of Spanish origin		124 (96.1)		92 (76.0)		151 (93.2)	
Race							
American Indian/Alaska Native		0		2 (1.7)		1 (0.6)	<0.001
Asian		7 (5.4)		23 (19.8)		5 (3.2)	
Black/African American		0		4 (3.4)		7 (4.4)	
Native Hawaiian/Pacific Islander		0		1 (0.9)		0	
White		122 (94.6)		85 (73.3)		145 (91.8)	
Biracial		0		1 (0.9)		0	
Clinical setting^c^							
Community Hospital		79 (59.8)		60 (47.6)		103 (62.0)	0.04
Academic Medical Center		46 (34.8)		35 (27.8)		13 (7.8)	<0.001
Private Practice		2 (1.5)		25 (19.8)		48 (28.9)	<0.001
Remote/telehealth		18 (14.0)		^b^		^b^	
Practice Outside the US		5 (3.8)		23 (18.3)		1 (0.6)	<0.001
Other		5 (3.8)		0		6 (3.6)	0.09
Years in practice							
Less than 1 year		11 (8.5)		3 (2.4)		4 (2.4)	<0.001
1–5 years		91 (70.6)		41 (32.8)		37 (22.3)	
6–10 years		20 (15.5)		18 (14.4)		45 (27.1)	
11–25 years		7 (5.4)		45 (36.0)		56 (33.7)	
More than 25 years		0		18 (14.4)		24 (14.5)	
Years as a cancer GC	3.6 (4.3)		–		–		
GCRA patients over career							
1–5		^b^		5 (4.7)		16 (11.4)	0.32
6–10		^b^		12 (11.2)		15 (10.7)	
11–25		^b^		15 (14.0)		18 (12.9)	
26–50		^b^		17 (15.9)		20 (14.3)	
51–100		^b^		39 (36.4)		56 (40.0)	
>100		^b^		19 (17.8)		15 (10.7)	
Practice focus							
Oncology		^b^		86 (68.3)		119 (71.7)	0.04
Genetics		^b^		13 (10.3)		3 (1.8)	
Primary care		^b^		3 (2.4)		5 (3.0)	
Surgery		^b^		8 (6.3)		14 (8.4)	
OB/GYN		^b^		6 (4.8)		9 (5.4)	
High risk cancer clinic		^b^		6 (4.8)		14 (8.4)	
Other		^b^		4 (3.2)		2 (1.2)	
Serve underserved population							
Yes		109 (85.2)		79 (66.4)		108 (69.2)	0.001
No		19 (14.8)		40 (33.6)		48 (30.8)	
Typical length of pre‐test session (min)	44.6 (12.8)		42.4 (22.4)		49.7 (18.9)		0.003

^a^ 15 respondents identified as “Other” were excluded from analysis here due to insufficient sample sizes for inclusion in hypothesis testing.

^b^ Data not available.

^c^ Multiple selections were possible. Percentages will not necessarily sum to 100%.

GCRA in Practice Survey**—**23 items were utilized from an 85‐item rating scale that measured clinician feedback about how adequately key elements of GCRA were incorporated into their practice, based on a comprehensive two‐visit counseling model. The 23 items assessed for this study addressed key components of the pre‐test consultation which was divided into 6 sections in the survey: (1) introduction/contracting with patient, (2) assessment and documentation of patient and family history, (3) discussion of basic principles of cancer genetics, (4) estimating mutation probabilities and empiric cancer risks, (5) genetic testing discussion and strategy, and (6) informed consent for clinical and/or research testing. Seventeen items, outlined in Table 2, were components that align with the essential elements of informed consent for genetic cancer risk assessment and testing recommended by the NSGC (Riley et al., 2012). The element, genetic information discrimination, was not assessed in this survey. Rating scale response choices were: *Adequately incorporated into my practice; Not adequately incorporated into my practice; Not part of my practice; Need more information about this activity/can't assess*. Each of the six sections also included an open‐ended prompt to identify barriers to delivering these elements of GCRA, totaling six open‐ended prompts. The survey was delivered during the 10th week of the course and took approximately 15 min to complete.

**TABLE 2:** Chi‐square tests of independence comparing adequate inclusion of elements of informed consent (*n* = 352–422).

Element of informed consent^a^	Component	Reported as addressed adequately (%)		*c* ^2^	df	Cramer's *V*	*p*
		Genetic counselor (*n* = 129)	Community clinician (*n* = 307)				
1) Purpose of the test and who to test	Identify the most appropriate individual(s) for testing	123 (97.6)	263 (88.9)	8.71	1	0.14	0.003
	Address who else might be at increased risk/benefit from testing	123 (97.6)	255 (88.9)	8.68	1	0.15	0.003
	Discuss rationale for the best person to test	123 (98.4)	282 (95.3)	2.36	1	0.08	0.13
2) General information about the gene(s)	Review of basic genetics	118 (95.9)	227 (81.4)	14.90	1	0.19	<0.001
	Review of cancer genetics	121 (96.0)	232 (82.3)	14.14	1	0.19	<0.001
	Describe features of hereditary cancer syndromes	112 (91.1)	225 (79.5)	8.11	1	0.14	0.004
3) Possible test results	Describe potential outcomes	125 (99.2)	272 (92.8)	7.20	1	0.13	0.01
4) Technical aspects and accuracy of the test	Explain the genetic testing process	124 (98.4)	281 (94.9)	2.77	1	0.08	0.10
	Describe test methods	62 (66.7)	197 (74.3)	2.03	1	0.08	0.16
	Address the risks, benefits, and limitations of testing	122 (97.6)	270 (92.2)	4.46	1	0.10	0.04
5) Economic considerations	Explain cost/turn‐around time/insurance coverage	119 (96.0)	250 (86.8)	7.78	1	0.14	0.01
6) Genetic information discrimination	Discuss implications of results on genetic discrimination	118 (95.9)	^b^	^b^	^b^	^b^	^b^
7) Psychosocial aspects	Identify potential contraindications for testing	90 (75.6)	172 (63.5)	5.55	1	0.12	0.02
	Assess patient's psychosocial support system	92 (77.3)	152 (65.2)	5.40	1	0.12	0.02
	Address psychological and ethical implications	103 (84.4)	229 (78.4)	1.95	1	0.16	0.16
8) Confidentiality	Protect autonomy, privacy, and confidentiality	115 (94.3)	291 (97.7)	3.09	1	0.09	0.08
9) Utilization of test results	Discuss importance of sharing information with at‐risk family members	125 (99.2)	275 (93.9)	5.83	1	0.12	0.02
10) Alternatives to genetic testing	Discuss alternatives for testing	94 (79.0)	255 (91.1)	11.12	1	0.17	0.001

^a^ Elements of informed consent outlined by the NSGC (Riley et al., 2012).

^b^ Data not available.

#### 2.3.2 Genetic counselor prospective survey

An anonymous electronic survey was created using Qualtrics Survey Software (September 2020). Thirty‐four questions from this 44‐question survey were utilized for this study. Questions included closed and open‐ended prompts related to eligibility, demographics, sources and purpose of referrals, pretest counseling behavior, rating scale questions about how adequately key elements of GCRA are incorporated into their practice, and barriers to incorporating elements of informed consent. The demographic and rating scale items analyzed from the retrospective Course Application and GCRA in Practice survey were utilized for the GC survey to allow comparison between the two study groups. The number of GCRA patients over career and practice focus were not assessed in the genetic counselor survey. One additional question was added to the GC survey to determine eligibility and 2 demographic questions were added to the GC survey to determine the age of respondents and years practicing as a cancer GC (Table 1). An additional rating scale item was also added to the GC survey to assess incorporation of the element of genetic information discrimination (Table 2). The GC survey also included the open‐ended prompt pertaining to identifying barriers of informed consent from the GCRA in Practice retrospective survey. This open‐ended prompt was only asked once in the GC survey, compared to six times in the GCRA in Practice survey. Five additional multiple‐choice questions, which were not in the retrospective survey, were developed to assess GC pre‐test counseling and referral behavior. The anonymous survey took approximately 15 min to complete.

### 2.4 Procedures

A quantitative and qualitative approach was used to explore cancer GCs' and community clinicians' facilitation of the informed consent process in their GCRA practice.

#### 2.4.1 Community clinicians

Retrospective data were collected from two surveys distributed through email to five cohorts of community clinicians enrolled in the intensive course between 2016 and 2020: a course application survey was distributed prior to course enrollment, and a GCRA in Practice survey was distributed during week 10 of the 13‐week course. Data were collected and managed using REDCap electronic data capture tool hosted at City of Hope (Harris et al., 2009, 2019).

#### 2.4.2 Genetic counselors

A prospective survey was distributed between September 2020 and December 2020 through the NSGC student research listserv; which was sent twice, with two reminders 2 weeks apart, for a total of four emails. Participants were also contacted through the American Board of Genetic Counseling research listserv, a call for participants was sent once per month for two consecutive months, for a total of two emails.

## 3 DATA ANALYSIS

### 3.1 Quantitative data analysis

Quantitative data were examined using SPSS (version 27) to ensure that responses met criteria for inclusion. Data were examined with histograms (for numeric variables) or frequency tables (for ordinal or categorical variables) to determine if respondents offered invariant responses. GCs were removed from the retrospective community clinician dataset to allow for comparison of community clinician and GC responses since they were surveyed separately. Providers not engaged in providing GCRA services at the time of data collection were excluded from both datasets. Respondents to the GC survey who declined to participate or provided no data after the first three questions were removed from the prospective GC survey dataset before analysis. Duplicate and incomplete responses were excluded.

Quantitative data from both surveys were combined and analyzed using SPSS Statistics (version 27). Chi‐square tests were run to compare the elements of informed consent that were adequately incorporated into the provider's practice by both populations: GCs and community clinicians. Descriptive statistics were utilized to assess demographic information from both participant populations, and pre‐test and referral behavior from the GC population. Pre‐test and referral behavior were not assessed for the community clinician population.

### 3.2 Qualitative data analysis

Three researchers, one practicing GC and two GC graduate students, independently conducted coding and iterative inductive content analysis of open‐ended questions assessing barriers to incorporating elements of informed consent into the pre‐test GCRA session (Vears & Gillam, 2022). Microsoft Excel was utilized to manage the data. Content categories were derived from the data, discussed, and compared. Content categories agreed upon by all three researchers were recorded. The frequency of responses coded under each category was tallied as outlined by Creswell, 2003.

## 4 RESULTS

Four hundred twenty‐four community clinicians were in the original retrospective dataset, after GCs and providers who were not engaged in providing GCRA services at the time of data collection were excluded; 307 community clinician responses remained to be analyzed.

One hundred fifty‐four cancer GCs responded to the prospective survey; 1 (0.6%) declined to participate in the study and 24 (15.6%) provided no data after the first three questions, and were removed from the dataset before analysis. Duplicate and incomplete responses were excluded, leaving 129 cancer GC responses to be analyzed. All responses were anonymous, and no identifying data was requested.

No erroneous data emerged from the analyses. Likewise, none of the respondents appeared to have offered invariant responses, and written responses to open‐ended questions appeared to be good‐faith efforts to provide valid answers.

### 4.1 Demographic characteristics of participants

A total of 436 participants participated in the study. One hundred and twenty‐nine cancer GCs participated in the prospective study, and 307 community clinicians participated in the retrospective review. The community clinician participants included 126 physicians, 166 physician assistants and advanced practice nurses, and 15 participants characterized who characterized themselves as “other”. The “other” category included certified nurse midwives, epidemiologists, researchers, and international clinicians who do not consider themselves physicians, PAs or APRNs. Participant characteristics are presented in Table 1.

Respondents were predominately female (96.9% GCs, 73.8% physicians, 98.8% APRNs and PAs), White (94.6% GCs, 73.3% physicians, 91.8% APRNs and PAs), and self‐identified as non‐Hispanic, Latino, or of Spanish origin (96.1% GCs, 76.0% physicians, 93.2% APRNs and PAs). The majority of community clinician respondents reported their practice focus to be in oncology (68.3% physicians and 71.7% APRNs and PAs), and although they had overall more years in practice than GCs, most reported seeing less than 100 GCRA patients throughout their career (82.2% physicians and 89.3% APRNs and PAs). The number of GCRA patients was not assessed for the GC respondents. The majority of all three cohorts served medically underserved populations as part or all of their practice (85.2% of GCs, 66.4% of physicians, 69.2% of APRNs and PAs), and 59.8% of GCs, 47.6% of physicians, and 62.0% of APRNs and PAs worked in community hospital settings. The typical length of a pre‐test session was similar among all participants (mean of 42.4–49.7 min).

### 4.2 Incorporation of elements of informed consent into practice

Both participant cohorts rated the degree to which key elements of the informed consent process are adequately incorporated into their practice. Results from the two cohorts were compared to determine which elements were similarly addressed by GCs and community clinicians and which elements differed (Table 2).

Five components corresponding with four elements of informed consent were reported by more than 90% of both GCs and community clinicians as well incorporated into practice (*p* > 0.05; Table 2): (1) discuss the rationale for the best person to test (purpose of the test and who to test), (2) explain the genetic process (technical aspects and accuracy of the test), (3) address the risks, benefits, and limitations of testing (technical aspects and accuracy of the test), (4) protect autonomy, privacy, and confidentiality (confidentiality), and (5) discuss importance of sharing information with at‐risk family members (utilization of test results).

Seven components corresponding with four elements of informed consent were more often reported as adequately addressed by GCs (Table 2): (1) identify the most appropriate individual(s) for genetic testing (*p* = 0.003; purpose of the test and who to test), (2) address who else might be at increased risk/benefit from testing (*p* = 0.003; purpose of the test and who to test), (3) review basic genetics (*p* = <0.001; general information about the gene(s)), (4) review cancer genetics (*p* = <0.001; general information about the gene(s)), (5) describe features of hereditary cancer syndromes (*p* = 0.004; general information about the gene(s)), (6) describe potential outcomes (*p* = 0.007; possible test results), and (7) explain cost, turn‐around time and insurance coverage (*p* = 0.005; economic considerations).

One component, discuss alternatives to genetic testing (alternatives to genetic testing), was significantly more often reported as adequately addressed by community clinicians (*p* = 0.001).

Although this GC population reported that they adequately incorporate more elements of informed consent into their pre‐test GCRA session than the community clinician population, the effect size between the two populations is small (Cramer's *V* = 0.10–0.20; Cohen, 1988).

The least adequately incorporated element of informed consent reported by both cohorts was the psychosocial aspects of testing, which included three components: identify potential contraindications for testing, assess a patient's psychosocial support system, and address psychological and ethical implications (psychosocial aspects).

### 4.3 Barriers to incorporating elements of informed consent into practice

Qualitative analysis of responses related to barriers to incorporating the elements of informed consent into practice revealed four key content categories: (1) time constraints, (2) lack of awareness, experience or education, (3) availability of resources or institutional support, and (4) patient‐related concerns. Table 3 summarizes and tallies these categories, along with exemplary quotes.

**TABLE 3:** Barriers to incorporating elements of informed consent into practice.

Reported barrier	Frequency of barrier reported *n* (%)^a^		Selected respondent quotes
	Genetic counselor (*n* = 129)	Community clinician (*n* = 307)	
Time constraints	16 (12.4)	164 (53.4)	Time with patients is my greatest barrier… I often am incorporating GCRA into clinical visits that are post‐operative where pathology, need for adjuvant treatment and recovery from surgery are all being discussed. (Community Clinician) Currently, there is a lack of time, structure and patient resources for this process. We will be addressing these issues. I selected ‘not adequately incorporated’ as all of these areas need improvement ‐ we do them, but obviously identified the need for improvement, which is why we are taking the course. (Community Clinician) I think the biggest barrier is time. It is difficult to spend time going through the psychological or psychosocial aspects of genetic testing with every single patient. In many ways, I have to triage these situations and know when this information is most important for this patient. (Genetic Counselor) Occasionally there will be time constraints if I am seeing a patient as part of a multidisciplinary clinic. In these cases, I do not skip over the most important parts of consent, but the patient may receive an abbreviated version… (Genetic Counselor)
Lack of awareness, experience, or education	0 (0.0)	81 (26.4)	Need more knowledge and experience regarding various cultural and psychosocial needs (Community Clinician) I have room to grow in being able to freely discuss the spectrum/penetrance of less common cancer syndromes. Physicians I work with can also use education with syndromes beyond HBOC and Lynch, but on a positive note, are very willing to learn and look up to ‘'cancer risk assessment experts’ to guide them in learning this information. (Community Clinician) As a provider, one big barrier is to continue education in this field to keep up with the fast development in this field. (Community Clinician) Just gaining more experience which would lead to increase confidence in my ability to relay information to my patients. (Community Clinician)
Availability of resources or institutional support	1 (0.1)	78 (25.4)	Time and staff! Usually during my 60‐minute appointment I get to know the patient, perform a breast exam, perform risk assessment based on calculations, discuss pre‐test of genetic testing, perform genetic testing, and set up appropriate imaging… I don't have the time or staff to get all medical records from family members and review all cultural/family dynamics. (Community Clinician) I work in a small, rural hospital where a Genetic Counselor sees patients 4 times annually. I would like to incorporate more genetic testing and counseling into my practice. We currently have several barriers to overcome before that could happen. We would need to have buy in from the local physicians to see patients with different genetic syndromes for close follow‐up. (Community Clinician) GCRA does not produce a stream of revenue for our clinic. Therefore, it is not a primary concern for the administration. Our office is committed to GCRA. However, it is difficult to expand our GCRA practice without the administrations blessing. (Community Clinician) Limited time to discuss basic genetics. It would be nice to have resources for patient education. (Community Clinician)
Patient related concerns	8 (6.2)	56 (18.2)	Some patients are not interested in that level of detail. In their mind, “the doctor I trust told me to get this testing done, so I am getting it done. Don't need to hear a whole genetics lesson” (Genetic Counselor) There is more of a pressure for genetic testing from the oncologists as it will impact treatment. Therefore, I believe many of these patients come to me already feeling pressured to have testing, so I have to “undo” the pressure. Additionally, many will defer to the opinion of the oncologist as they do often see GC as non‐entity. (Genetic Counselor) Patient baseline knowledge of genetics is often very limited; it would be helpful to learn how to communicate complex genetic concepts at a low education level. (Community Clinician) I tend not to focus too much on the testing process, because patients usually just glaze over. I summarize things to which level a patient may be at. (Community Clinician) I think many of the sessions are information overload for the patient, so I don't want to cover too much if they're overwhelmed. (Genetic Counselor) All of this varies depending on patient situation, baseline education, medical knowledge. (Community Clinician) Some barriers are education: if a patient has low health literacy, it takes more time to go through all these, and so the sessions are longer, and I need to end in 60 minutes to see the next patient. (Genetic Counselor) A lot of my patients do not have insurance or funds to cover genetic testing. (Community Clinician)
No barriers reported	104 (80.6)	21 (6.8)	

^a^ Multiple coded content categories were possible. Percentages will not necessarily sum to 100%.

The most consistent barrier reported across disciplines was time constraints. Several community clinicians reported that they do not solely provide pre‐test counseling services at the time of the patient appointment, which means they are only spending a fraction of a typical appointment on GCRA. The primary aspect of the informed consent process that GCs and community clinicians reported excluding due to time barriers was addressing psychosocial concerns. According to clinicians, this included patient readiness for testing, possible repercussions of a positive result, and the overall psychological impact that genetic testing may have on a patient's mental well‐being. Several GCs also outlined the importance of abbreviating this information when time is limited.

Patient‐related concerns were also reported by both CCs and GCs as a challenge to overcome. These include financial barriers such as insurance coverage, along with patient engagement, interest, preconceived notions about testing, and baseline education level.

Providers' lack of awareness of the elements of informed consent was another frequently reported barrier. Secondary to lacking awareness, several community clinicians reflected that they did not have enough experience to incorporate advanced GCRA skills into their practice, leading to a lack of comfort and confidence facilitating informed consent. Some community clinicians also expressed their desire to broaden the application of GCRA in their practice, but their lack of knowledge in certain aspects of informed consent kept them from expansion. As stated by a physician “I felt I needed a more comprehensive education before moving into areas beyond HBOC [hereditary breast and ovarian cancer] testing, and even to expand my understanding of how to address the changes in HBOC in recent years.*”*

Community clinicians reported the limited availability of resources at their disposal being a barrier. The three most reported limited resources were psychosocial resources, institutional financial resources, and support staff. Another factor reported to influence the availability of resources is the lack of institutional support for the community clinician's GCRA practice. Due to this constraint, several community clinicians reported that they do not have enough support staff to sustain their GCRA practice. Community clinicians noted that this lack of administrative support staff makes it more challenging to complete additional tasks such as obtaining relevant patient records.

## 5 DISCUSSION

Ongoing advances in genetic and genomic technologies, awareness of the utility of cancer genetic testing for prevention and treatment, and the shortage of clinicians with advanced degrees in genetics, continue to drive the demand for alternative approaches to scale up the delivery of GCRA services. With a larger breadth of providers ordering genetic testing and alternative service delivery models being developed to meet the demand for testing, it is imperative that we understand if the essential elements of informed consent are being addressed prior to genetic testing, and the barriers providers face to facilitating this process.

The purpose of this study was to gather perspectives and experiences with facilitating the informed consent process from GCs and community clinicians including physicians, physician assistants and advanced practice registered nurses who are actively engaged in delivering GCRA services in diverse practice settings. To our knowledge, this is the first time that providers' practice in the informed consent process in genetic cancer risk assessment and barriers faced to facilitating this process have been studied.

Our study found that more than 90% of GCs and community clinicians consistently and adequately reported that they addressed 5 out of the 10 elements of informed consent outlined by the NSGC (Riley et al., 2012) during a pre‐test GCRA session, with technical aspects and accuracy of the test and utilization of test results being the most similarly addressed elements in both cohorts. The least addressed element reported by both cohorts was psychosocial aspects of the informed consent process (Table 2).

### 5.1 Practice implications

Overall, the GCs and community clinicians in our study reported that they adequately addressed several key elements of informed consent with their patients. However, they also described several factors that may cause them to eliminate or abbreviate some elements of the pre‐test informed consent process (Table 3). The primary barrier reported by 180 respondents was time constraints. This finding aligns with additional studies which found that both providers and patients view time as a major barrier to care, with the greatest impact of this barrier being on the shared decision‐making process (Légaré et al., 2008; Yahanda & Mozersky, 2020). The facilitation of informed consent includes the process of collaborative decision making where the patient has autonomy over their decision (Samuel et al., 2017). Given the impact of time on the informed consent process revealed in this study, it is reasonable to assume that the barrier of time constraints could impact the optimal facilitation of collaborative decision making during the pre‐test GCRA session.

Community clinicians and GCs reported that, when faced with time constraints within a pre‐test session, they typically focus on what they believe are the most critical aspects of the informed consent process (Table 3). Confidentiality was one element of informed consent that was well incorporated into practice with 97.7% of community clinicians and 94.3% of GCs reporting that they adequately address protecting autonomy, privacy, and confidentiality (Table 2). While patient autonomy was noted as a priority for both community clinicians and GCs, some reported a challenge ensuring that testing is the patient's decision in instances when patients are told by their referring provider that they need to complete testing to help guide treatment decisions (Table 3). This is to be expected in GCRA, as germline genetic testing is increasingly used to help guide treatment decisions including targeted therapies and enrollment in clinical trials for individuals with a diagnosis of cancer (Domchek et al., 2020; Stadler et al., 2021; Yap et al., 2021). Some respondents also reported patients' preconceived expectations as a barrier, as noted by a PA respondent, “Most patients referred for GCRA are already anticipating being tested. It is difficult to explain to a patient that they may not need any type of testing.”

In our study another of the most frequently addressed elements of informed consent was possible test results (positive, variant of uncertain significance (VUS), and negative) with 99.2% of GCs and 92.8.% of community clinicians stating that they adequately addressed these possible outcomes with patients prior to undergoing genetic testing (Table 2). Understanding these possible results prior to testing, particularly in the case of a VUS, could impact perception of risk and subsequent medical decisions (Culver et al., 2013; Idos et al., 2019). Previous studies found that physicians have varying levels of knowledge about the possible test results, with oncologists being the most well versed (Hauser et al., 2018; Macklin et al., 2019).

Psychosocial aspects were reported as the least addressed element of the informed consent process, with 20–40% of both cohorts reporting that they may not adequately assess a patient's psychosocial support system and identify potential contraindications for testing (Table 2). This information was strongly reflected in the open‐ended responses. Some respondents shared that limited time is a barrier to conducting a thorough psychological assessment as illustrated by a comment from an APRN respondent, “It is a difficult in a short period of time to assess family dynamics, psychosocial support, cultural and religious beliefs and perception of cancer,” and a physician respondent, “Inadequate time to really explore the psychosocial components beyond a typical new patient intake of their history.” Both cohorts reported that they do their best to determine if their patients may need psychosocial support, and if not, the psychosocial aspects of informed consent is an element they will abbreviate or eliminate to complete the session within the allotted time frame. One GC explained that it is often less incorporated given directiveness of referring providers: “I hesitate to say that addressing psychological factors and a patient's psychosocial support system are not adequately incorporated in my pre‐test session…these can often be difficult to fully address because patients have been told by another provider that they should do testing, regardless of their psychological readiness for it. Therefore, although I may assess that a patient is psychologically fragile, they would not be receptive to my discussion regarding the psychological ‘risks’ to them.” Some community clinicians report that their lack of knowledge and awareness of this element of the informed consent process may impact whether it is adequately incorporated into their practice. As noted by an APRN, their new awareness and knowledge of this element after completing the educational course will allow them to incorporate more psychosocial counseling into their practice, however the lack of trained social workers will still be a barrier: “I now have a heightened awareness of psychosocial impact…I hope this will help me to better identify depression, psychosocial problems and make necessary referrals when indicated. A barrier in our institution is the lack of dedicated oncology social workers.” In many instances both cohorts reported that, if they see a need, they refer to a social worker or psychologist. Some clinicians reported not having the ability to refer due to lack of accessibility of psychological specialists. This coincides with the community clinician reported barrier of the availability of resources and institutional support. Lack of resources and institutional support were not reported to be a barrier for GCs (Table 3), although some studies do note this to be a barrier for GCs (Boothe et al., 2021; “NSGC Professional Status Survey”, 2023).

Overall, our findings suggest that GCs incorporate more elements of informed consent into their GCRA sessions than community clinicians (Table 2). The elements reported as most often addressed were purpose of the test and who to test (identify the most appropriate individual(s) for testing and address who else might be at increased risk/benefit from testing), general information about the gene(s) (review of basic genetics, review of cancer genetics and describe features of hereditary cancer syndromes) and economic considerations (explain cost, turn‐around‐time, and insurance coverage). These statistically significant differences between the two cohorts may be due to the specialized education and training GCs have in genomics and counseling, compared to the varying levels of genomics education among community clinicians (Carroll et al., 2016; Plunkett‐Rondeau et al., 2015). As noted by a physician respondent, “My lack of knowledge and my time constraints. Penetrance, etc were things briefly reviewed in med school 25 years ago!! I had forgotten much of my genetics. Better now…Time will always be an issue until we have dedicated personnel.” This was also reflected in feedback from one APRN, “I feel most of the activities that aren't incorporated are because of lack of experience. Being able to listen to other counseling sessions would be helpful to learn how they incorporate these things.” It is also possible that community clinicians have different priorities while facilitating the informed consent process, given that some stated that they often incorporate pre‐test GCRA into a typical clinical visit with other primary responsibilities such as cancer screening, treatment, and management (Table 3).

Regardless of the reason for incorporation or lack thereof, a subtheme that was present was the value and need for continuing education to increase awareness, knowledge, and confidence in order to better address all the necessary elements of the informed consent process. “I have learned a lot from this course but need a little time for some confidence building about what I have learned,” responded one APRN. A physician respondent noted, “I expect that at the completion I will be more knowledgeable…and just gaining more experience would lead to increase confidence in my ability to relay information to my patients…if I feel they are at risk, send them to our genetic counselor…once I have completed the course I may be able to have the full counseling session with the patient myself, order the appropriate test and conduct the posttest discussion myself.” This finding is also reflected in a previous study that assessed physicians' understanding of cancer genomics and educational models (Weipert et al., 2018).

Education on the process of informed consent is vital for community clinicians, given that only 42% of GCs reported that they conduct pre‐test GCRA for all their patients (Table S1). In instances where they are not conducting pre‐test GCRA and facilitating the process of informed consent, additional findings from our study revealed that most of the remaining pre‐test counseling is conducted by medical and surgical oncologists, primary care providers, and OB/GYNs (Table S1). This information, paired with the shortage of genetics experts and the ongoing evolution of alternative service delivery models, reinforces the need for ongoing collaboration between community clinicians and GCs (Stoll et al., 2018). This collaboration was reflected in the qualitative data with several GCs and community clinicians agreeing that collaboration between specialties helps combat barriers faced: “Biggest barrier is time; however, I am blessed to have excellent genetic and psychosocial counselors to help realize these important aspects of GCRA,” noted a physician respondent. In fact, a prevailing subtheme of the study was community clinicians' motivation to learn about genetics and desire to adapt to the needs of their patients, as expressed by an APRN, “I have room to grow in being able to freely discuss the spectrum/penetrance of less common cancer syndromes. Physicians I work with can also use education with syndromes beyond HBOC and Lynch, but on a positive note, are very willing to learn and look up to ‘cancer risk assessment experts’ to guide them in learning this information.”

Community clinicians and GCs reported several patient‐related concerns that may impact the informed consent process. These included patient baseline educational level, patient priorities, understanding of the utility of genetic counseling and testing, and accessibility of information (Table 3). Both community clinicians and GCs reported that quality patient educational materials and resources would be beneficial to help address many of these barriers. As they have “limited time to discuss basic genetics…would be nice to have resources for patient education,” and “…sufficient materials to aid in these educational discussions,” noted two physician respondents. Several community clinicians and GCs in this study stated that patient educational materials are necessary as they may help mitigate patients' preconceived notions about genetic testing. This is supported by several studies that have assessed the readability of patient‐centered cancer genetics materials and found that most materials were not suited for the lay person (Azer et al., 2018; Brown et al., 2011; Keinki et al., 2018; Parker et al., 2022). Increasing the readability of these materials may also increase accessibility of genetic services to underserved populations (Kaphingst et al., 2015).

Several other clinicians noted that the barrier of baseline patient education level may be in part due to the way information is presented to patients, as it is difficult to tailor often‐complex genetic concepts to the patient's needs. Continuing provider education was suggested to assist in combatting some of the reported patient barriers, as one APRN stated, “patient baseline knowledge of genetics is often very limited, it would be helpful to learn how to communicate complex genetic concepts at a low education level.” Referring provider education about the utility of genetic testing process was also noted by one APRN to a be a barrier, “physician education ‐ some patients come for testing only because their physician told them they needed it.” Given that 82% of referrals to GCs in our study are for GCRA, and these referrals primarily are from medical and surgical oncologists, OB/GYNs, and primary care providers (Figures S1 and S2), it is important to create more opportunities to educate community clinicians about the informed consent process, as this will assist them in setting expectations for their patients before referring them for GCRA.

### 5.2 Study limitations

Retrospective study participants were from a small subset of the community clinician population. Their enrollment in the CCGCoP course depicts their motivation to learn more about GCRA and improve their practice. Additionally, 100% of community clinicians included in this study have conducted GCRA and ordered genetic testing. Therefore, this community clinician cohort may not be representative of the broader community clinician population. The clinicians that formed part of this study also included international and domestic community clinicians and GCs. Roles, responsibilities, and resources may differ between international and domestic providers.

Although the community clinician cohort had overall more years in practice than the GC cohort, most participants in the community clinician cohort had conducted fewer than 100 GCRA appointments throughout their career (Table 1). The number of GCRA appointments conducted by GCs was not captured in this study, however, the 2023 NSGC professional status survey found that cancer GCs conduct a median of 50 GCRA appointments per month (“NSGC Professional Status Survey”, 2023). Given that most GC respondents have been practicing for 1 to 5 years, it is feasible that the GC respondents in our study have seen more than 100 GCRA patients throughout their career. Additionally, the community clinicians, although they had conducted GCRA appointments, were enrolled in the course in an effort to gain proficiency in GCRA. The surveys administered to the community clinicians were conducted during their participation in the course, therefore they had not completed their specialized training in GCRA. Conversely, the GCs in the study had completed their specialized Master's education in genetic counseling at the time of survey distribution. It is possible that the overall lower rate of adequately addressing the elements of informed consent reported in Table 2 by community clinicians may be due to their lack of awareness of the elements of informed consent at the time of survey distribution. Lack of awareness was also noted to be a barrier by community clinicians (Table 3). Furthermore, it is important to note that for both cohorts, clinicians self‐reported that they adequately addressed elements of informed consent in their GCRA practice. As we did not provide an operational definition of the term ‘adequate’ in the survey, respondents may have defined the term ‘adequate’ differently.

Community clinicians reported that they often provide GCRA within a standard appointment's time frame; therefore, the entire appointment may not be dedicated solely to GCRA (Table 3). This may have significance regarding the time they reported spending in a pre‐test consultation (Table 1). Although GCs and community clinicians reported spending a similar amount of time on a pre‐test consultation, it is possible that overall GCs spend more time dedicated to GCRA and the facilitation of informed consent. Additionally, community clinicians have areas of expertise that may aid in their pre‐test GRCA, that GCs do not. For example, they may conduct a physical examination for clinical features of hereditary cancer syndromes that would aid in differential building and decision making. This data was collected as part of the GCRA in Practice survey, but these areas of expertise were not assessed in this study.

Furthermore, the community clinician surveys asked about ELSI and communication, however, they did not assess for the genetic discrimination element of informed consent. For this reason, it was not possible to determine if community clinicians incorporate this element into practice. Studies do show that this is an area of the informed consent process that some providers do not feel comfortable addressing (Giri et al., 2022; Hauser et al., 2018). This was not a prevailing content category; however, a few community clinicians did cite this as a barrier to their GCRA practice, “I have never felt like I can adequately address questions regarding job/insurance discrimination,” stated a physician respondent.

### 5.3 Future directions

Alternative service delivery models are continuing to be developed to mitigate the barriers of time constraints and assist in meeting the increased demand for genetic testing. Although these models have proven to be effective to increase uptake of genetic testing, many of the models rely on providers, especially those in community settings, to be trained to triage patients, provide pre‐test counseling, and order genetic testing (Furniss et al., 2021; Ormond et al., 2019; Robson et al., 2015). In addition to models that rely heavily on collaboration between providers, several models rely on web‐based learning and the development of educational tools targeted to patients to help supplement or replace the informed consent process facilitated by providers (Campion et al., 2019; Cohen et al., 2012; Loeb et al., 2022; McCuaig et al., 2018). The information learned from this study may be utilized to inform the development of these models.

An important aspect of facilitating the informed consent process is collaboration between provider and patient, and between community clinicians and providers with formal genetics training. One physician explained how they collaborate with a GC, “Right now…if I feel they are at risk, [I] send them to our genetic counselor. It is anticipated that at least for breast patients, once I have completed that course that I may be able to have the full counseling session with the patient myself, order the appropriate test and conduct the posttest discussion myself.” This quote also depicts the value of continuing education to build confidence. Currently, the primary forms of continuing education in genomic medicine are electronic and web‐based learning, interdisciplinary and interprofessional education, and immersive and experiential learning (Rubanovich et al., 2018). Although continuing education resources exist, more research needs to be conducted on the efficacy of continuing educational tools for clinicians to assist them in facilitating informed consent in GCRA. In our study, community clinicians expressed both their desire for quality educational tools in cancer genomics and increased access to GCs. Increased provider education may lead to a more collaborative team‐based approach to GCRA, help in meeting the demand for genetic testing, and support the implementation of alternative service delivery models that rely on community clinicians to take part in the GCRA process. Findings from this study may be utilized to develop these continuing educational tools. For example, given the importance of the potential outcomes of testing reported by both cohorts and the varying levels of provider understanding of these outcomes that studies report, it may be beneficial to develop continuing education resources for providers with a focus on the possible test results and supplemental educational tools for patients outlining the possible test results (Hauser et al., 2018; Macklin et al., 2019). Additionally, this study focused on providers who were actively participating in specialized education for GCRA, which is only a small subset of providers who order genetic testing for hereditary cancer risk. Initiatives to further genetics education for community providers across disciplines is needed to ensure that all providers who deliver GCRA understand the elements of informed consent that should be addressed prior to testing. These initiatives may include educational tools that are integrated into continuing education and easily accessible practice guidelines and policy statements to keep up with the rapid advancements in the field.

Regardless of whether all community providers were educated on the elements of informed consent, time will continue to be a barrier to facilitating this process. For this reason, the development of quality targeted supplemental educational tools for patients to utilize outside of the pre‐test appointment would be beneficial to mitigate this barrier and ensure patients are able to make an informed decision about pursuing genetic testing. This includes continued development of targeted patient educational tools and resources for clinicians to help convey complex genetic concepts to patients as this may help bridge the barriers of patient educational level and lack of awareness of genetic services that were reported (Table 3). These patient educational tools may be geared towards the elements of informed consent not adequately addressed by providers (Table 2). Additionally, it would be beneficial to conduct studies that assess which educational tools patients find most valuable and effective. It may also be useful to survey patients who have already received genetic testing to determine the elements of informed consent they believe were most important or wish had been discussed in more detail when trying to make an educated decision about testing.

Of note, the NSGC practice resource outlining the ten essential elements of informed consent for genetic testing in cancer genomics was published in 2012, prior to the introduction of multi‐gene panel testing and integration of alternative delivery models into the field of clinical cancer genetics (Riley et al., 2012). Other studies have suggested a revised approach to informed consent informed by the complexities of evolving genetic testing technologies and models of care (Bradbury et al., 2015; Koplin et al., 2022; Schienda & Stopfer, 2020). These efforts and findings of our study suggest that a re‐review of the current NSGC informed consent guidelines may be warranted.

## 6 CONCLUSION

The findings of this study supported that both GCs and community clinicians agree that the elements of informed consent are important to address prior to testing. However, they encountered barriers that restricted their ability to facilitate all elements of the informed consent process. It is important that all clinicians engaged in pre‐test GCRA be aware of and educated about the elements of informed consent. This may warrant additional educational opportunities for clinicians to keep up with the rapid advancements in the field and to increase their awareness and confidence in facilitating the informed consent process. As we shift to alternative service delivery models which allow providers to abbreviate or eliminate their role in the facilitation of informed consent, efforts should be made to ensure that the essential elements of the informed consent process are retained. One approach to achieving this is to create relevant, easily accessible resources for providers across disciplines through practice guidelines and policy statements. Additionally, alternative delivery models should be developed to incorporate the elements of informed consent regardless of the avenue of delivery. Furthermore, by understanding which elements of informed consent are addressed in a typical pre‐test session, supplementary educational tools for patients may be geared towards the elements that providers often have to eliminate or abbreviate in a session.

## AUTHOR CONTRIBUTIONS

Author Alexandra Capasso confirms that they had full access to all the data in the study and take responsibility for the integrity of the data and the accuracy of the data analysis. All of the authors gave final approval of this version to be published and agree to be accountable for all aspects of the work in ensuring that questions related to the accuracy or integrity of any part of the work are appropriately investigated and resolved.

## CONFLICT OF INTEREST STATEMENT

Authors Alexandra Capasso, Bita Nehoray, Nicholas Gorman, Emily Quinn, Daiana Buccio, and Kathleen Blazer declare that they have no conflict of interest.

## ETHICS STATEMENT

Human studies and informed consent: This study was reviewed and granted an exemption by the *Institutional Review Board of Claremont Graduate University (IRB # 3838)*. All procedures followed were in accordance with the ethical standards of the responsible committee on human experimentation (institutional and national) and with the Helsinki Declaration of 1975, as revised in 2000. Implied informed consent was obtained for individuals who voluntarily completed the online survey and submitted their responses.

Animal studies: No non‐human animal studies were carried out by the authors for this article.

## Supporting information


Data S1.

## Data Availability

The data that support the findings of this study are available within the article.
