# Supplementary material for: Genetic counselors' and community clinicians' implementation and perceived barriers to informed consent during pre‐test counseling for hereditary cancer risk
Source: J Genet Couns. 2024 Mar 13;34(1):e1887. doi: 10.1002/jgc4.1887 (PMC11393174; doi:10.1002/jgc4.1887)
Supplement: Supplementary file 1 — Data S1. [file JGC4-34-0-s001.zip › Manuscript Supplementary Information Files Revised_ACapasso.docx]

Supplemental Data

Tables

*Table 4. Providers Performing Pre-test Counseling when not Performed by a Genetic Counselor (n = 129)*

| **Response** |  | **Valid %** |
| --- | --- | --- |
| N/A Provide Pre-test Counseling for All Patients |  | 41.9% |
| Medical Oncologist |  | 27.1% |
| OB/GYN |  | 21.7% |
| Surgical Oncologist |  | 20.1% |
| Primary Care |  | 17.8% |
| N/A: Patients Did Not Receive Pre-test Counseling |  | 16.3% |
| Advanced Practice Registered Nurse |  | 10.1% |
| Other |  | 10.1% |
| GC within Your Practice |  | 8.5% |
| N/A Direct Access Testing |  | 8.5% |
| GC Outside Your Practice |  | 7.8% |
| Telehealth Genetic Counseling |  | 7.0% |
| GC from a Lab |  | 4.7% |
| Imaging Center |  | 3.9% |
| Medical Geneticist |  | 3.1% |
| Nurse Midwife |  | 3.1% |

Less than half of genetic counselors provide pre-test counseling for all of their patients. When pre-test counseling is not performed by a genetic counselor, patients are most often receiving pretest GCRA by their medical or surgical oncologist, OB/GYN, or primary care clinician. Nearly a quarter of patients do not receive pretest counseling at all, in part due to having had direct access testing.

Figures

*Figure 2. Genetic Counseling Referring Providers (n = 129)*


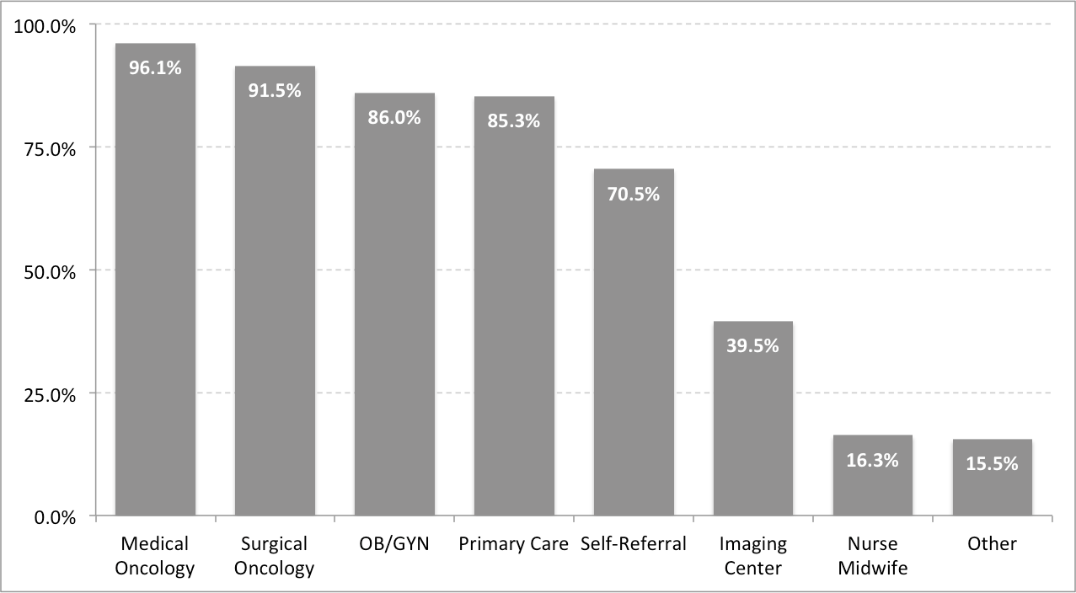


The majority of referrals come from medical and surgical oncologists, OB/GYNs and primary care providers. Cancer GCs also receive self-referrals. ‘Other’ included gastroenterology, naturopaths, endocrinology, dermatology, ophthalmology, and fertility clinics.

*Figure 3. Purpose of Genetic Counseling Referrals (n = 129)*


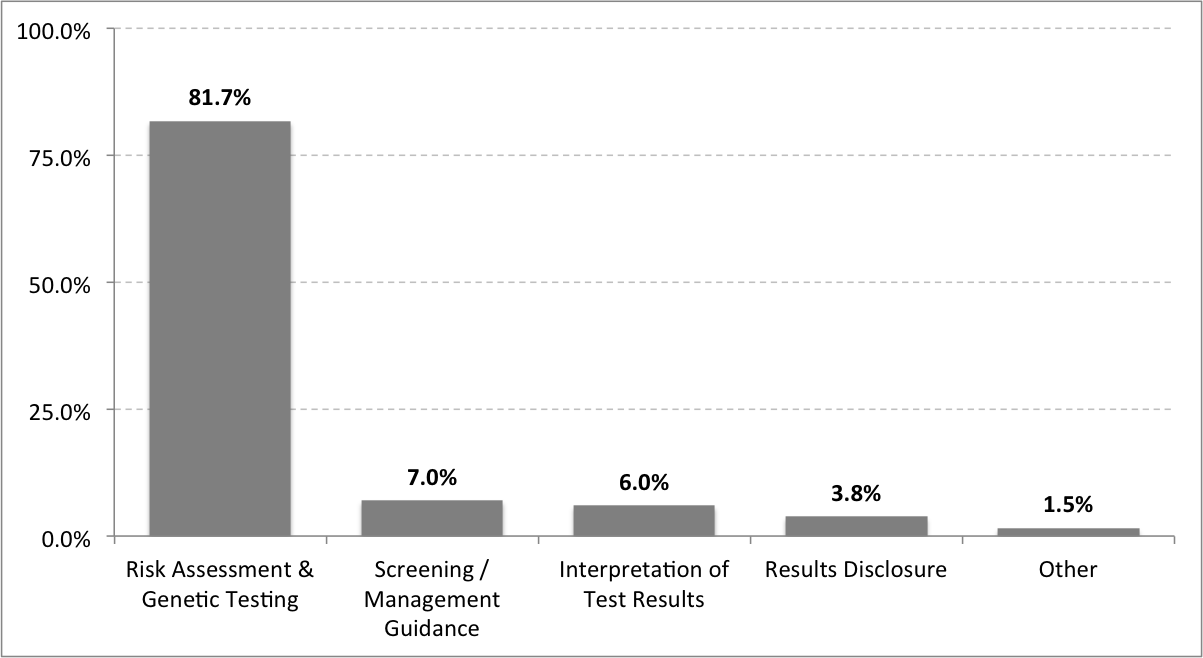


The vast majority of referrals to genetic counselors were for risk assessment and genetic testing. Rarely were patients referred for other reasons.
