# Supplementary material for: Genetic counselors' and community clinicians' implementation and perceived barriers to informed consent during pre‐test counseling for hereditary cancer risk
Source: J Genet Couns. 2024 Mar 13;34(1):e1887. doi: 10.1002/jgc4.1887 (PMC11393174; doi:10.1002/jgc4.1887)
Supplement: Supplementary file 1 — Data S1. [file JGC4-34-0-s001.zip › Survey Instruments Supplemental.docx]

**Survey Instruments**

Only the survey items utilized in the study are included.

**Genetic Counselor Survey Instrument**

**Eligibility**

1. Are you a cancer genetic counselor?

- Yes
- No (skip to end of survey)

1. How many years of experience do you have practicing as a genetic counselor? _________
2. How many years of experience do you have practicing as a clinical cancer genetic counselor? _________

**Demographics**

1. Age _______
2. Sex

- Male
- Female
- Other (please specify:_________________)

1. Are you of Hispanic, Latino or Spanish origin?

- Yes
- No

1. What is your race? (check all that apply)

- American Indian or Alaska native
- Asian
- Black or African American
- Native Hawaiian or Pacific Islander
- White
- Other (please specify:___________)

1. What type of setting do you practice in? (check all that apply)

- Community Hospital (including private, academic-affiliated, other)
- Academic Medical Center
- Private Practice
- Remote/telehealth
- Practice outside the US
- Other (please specify:_______________)

1. Does all or part of your practice serve a minority/socioeconomically underserved population?
   - Yes
   - No
2. Where does your practice receive referrals from? (check all that apply)

- Primary care
- OB/GYN
- Medical oncology
- Surgical oncology
- Imaging center
- Nurse Midwife
- Self-referral
- Other (please specify:_______________)

1. What is the purpose for these referrals? Please assign a percentage to each option below, total should add up to 100%

- Risk assessment and genetic testing____%
- Results disclosure_____%
- Interpretation of test results after disclosure by another provider_____%
- Screening/management guidance_____%
- Other _____% (please specify:_____________________)

1. Do you conduct pre-test counseling?

- Yes
- No

1. In circumstances where you do not provide pre-test counseling, please indicate the reasons why? (select all that apply)
   - Time constraints
   - Limited genetics personal
   - Limited financial resources
   - Not within the scope of the practice
   - Not interested in pre-test counseling
   - Other (please specify:______________)
   - N/A
2. In circumstances where you do not provide pre-test counseling, where do your patients receive pre-test counseling? (check all that apply)

- Primary care
- OB/GYN
- Medical oncologist
- Surgical oncologist
- Imaging center
- Nurse practitioner
- Nurse midwife
- Medical geneticist
- Genetic counselor within your current practice
- Genetic counselor outside of your current practice
- Telehealth genetic counselor/genetic counseling service
- Genetic counselor from a lab
- N/A direct access testing
- N/A patients did not receive pre-test counseling
- Other (please specify:___________________)

1. Please rate the following elements of informed consent in a pre-test counseling session based upon your experience practicing as a clinical cancer genetic counselor

|  | Adequately incorporated into my practice | | Not adequately incorporated into my practice | Not part of my practice | Need more information about this activity/can’t assess |  |
| --- | --- | --- | --- | --- | --- | --- |
| 1. Identify the most appropriate individual(s) for genetic testing | | |  |  |  |  |
| 2. Address who else might be at increased risk/benefit from testing | | |  |  |  |  |
| 3. Discuss rationale for the best person to test | | |  |  |  |  |
| 4. Review of basic genetics (genes, chromosomes, inheritance patterns) | | |  |  |  |  |
| 5. Review of cancer genetics (sporadic, familial, hereditary, mechanisms of tumorigenesis) | | |  |  |  |  |
| 6. Describe features of hereditary cancer syndromes (spectrum, penetrance, etc.) | | |  |  |  |  |
| 7. Describe potential outcomes (positive, true negative vs. uninformative) | | |  |  |  |  |
| 8. Explain the genetic testing process | | |  |  |  |  |
| 9. Describe test methods (e.g., techniques, research vs. clinical testing) | | |  |  |  |  |
| 10. Address the risks, benefits, and limitations of genetic testing | | |  |  |  |  |
| 11. Explain cost/turn-around time/insurance coverage issues | | |  |  |  |  |
| 12. Identify potential contraindications for genetic testing (depression, other significant psychosocial problems) | | |  |  |  |  |
| 13. Assess patient's psychosocial support system | | |  |  |  |  |
| 14. Address psychological, ethical implications | | |  |  |  |  |
| 15. Protect autonomy/privacy/confidentiality | | |  |  |  |  |
| 1. Discuss implications of genetic test results on genetic discrimination | | |  |  |  |  |
| 17. Discuss importance of sharing information with at-risk family members | | |  |  |  |  |
| 18. Discuss alternatives to testing (e.g., empiric risk) | | |  |  |  |  |

1. Please describe barriers related to incorporating any of the above activities you consider important that are not part of your practice ______________________________________________________________________________________________________________________________________________________
2. What is the typical length (in minutes) of your pre-test session? _______

**Community Clinician Survey Instruments**

**Application**

1. PRIMARY Clinical Credentials
   - Physician
   - PhD
   - Nurse (all categories)
   - Genetic Counselor (Masters in Genetic Counseling required)
   - Physician’s Assistant
   - Other (please specify:___________________)

1a. Secondary Clinical Credentials (if applicable)

- - None
  - Physician
  - PhD
  - Nurse (all categories)
  - Genetic Counselor (Masters in Genetic Counseling required)
  - Physician’s Assistant
  - Other (please specify:___________________)

1b. Specific professional credentials (e.g., MD, PhD): _________________

1. Specialty Certification(s): (Check all that apply)
   - Oncology
   - Surgery
   - OB/GYN
   - Clinical Genetics
   - Family Practice
   - Genetic Counseling
   - Other (please specify:___________________)
2. Practice Focus
   - Oncology
   - Genetics
   - Primary care
   - Other (please specify:___________________)
3. Gender
   - Male
   - Female
   - I prefer not to answer
4. Race (select all that apply)
   - American Indian or Alaska Native
   - Asian
   - Black or African American
   - Native Hawaiian/Other Pacific Islander
   - White
   - I prefer not to answer
5. Ethnicity
   - Hispanic/Latino
   - Not Hispanic/Latino
   - I prefer not to answer
6. Primary designation of your practice setting(s):
   - Community Hospital
   - Community Hospital with Academic Affiliation
   - Private Practice
   - Academic Institution
   - Industry
   - Other practice setting designation (please specify): (e.g. military/veteran hospital)____________________

7a. Is your primary practice outside of the US?

- - Yes
  - No

1. Number of years you have been in clinical practice
   - N/A
   - Less than 1 year
   - 1 to 5 years
   - 6 to 10 years
   - 11 to 25 years
   - More than 25 years

8a. Does all or part of your clinical practice serve a minority/socioeconomically underserved population?

- - No/Not Applicable
  - Yes

1. Do you currently provide genetic cancer risk assessment (GCRA) services (genetic counseling and genetic testing for cancer predisposition)?
   - Yes, I am currently providing GCRA services
   - No, I have never provided GCRA services
   - I have provided GCRA services in the past, but I am not currently providing these services
2. Approximately how many patients have you provided GCRA services to over your career:
   - None
   - 1 to 10
   - 11 to 25
   - 26 to 50
   - 51 to 100
   - 101 to 500
   - More than 500
3. What is the typical length (in minutes) of the PRE-TEST GCRA session in your current practice? ___________________

**GCRA in Practice Survey**

Initial GCRA Consultation:

The following questions pertain to Introduction and Contracting with Patient.

1. Identify potential contraindications for genetic testing (depression, other significant psychosocial problems
   - Adequately incorporated into my practice
   - Not adequately incorporated into my practice
   - Not part of my practice
   - Need more information about this activity/can’t assess
2. Please describe barriers related to incorporating any of the above activities you consider important that are not part of your practice. _________________________

The following questions pertain to Assessment and Documentation of Patient and Family History.

1. Assess patient's psychosocial support system
   - Adequately incorporated into my practice
   - Not adequately incorporated into my practice
   - Not part of my practice
   - Need more information about this activity/can’t assess
2. Please describe barriers related to incorporating any of the above activities you consider important that are not part of your practice. _________________________

The following questions pertain to Discussion of Basic Principles of Cancer Genetics.

1. Review of basic genetics (genes, chromosomes, inheritance patterns)
   - Adequately incorporated into my practice
   - Not adequately incorporated into my practice
   - Not part of my practice
   - Need more information about this activity/can’t assess
2. Review of cancer genetics (sporadic, familial, hereditary, mechanisms of tumorigenesis)
   - Adequately incorporated into my practice
   - Not adequately incorporated into my practice
   - Not part of my practice
   - Need more information about this activity/can’t assess
3. Describe features of hereditary cancer syndromes (spectrum, penetrance, etc.)
   - Adequately incorporated into my practice
   - Not adequately incorporated into my practice
   - Not part of my practice
   - Need more information about this activity/can’t assess
4. Please describe barriers related to incorporating any of the above activities you consider important that are not part of your practice. _________________________

The following questions pertain to Estimating Mutation Probability and Empiric Cancer Risks.

1. Identify the most appropriate individual(s) for genetic testing
   - Adequately incorporated into my practice
   - Not adequately incorporated into my practice
   - Not part of my practice
   - Need more information about this activity/can’t assess
2. Address who else might be at increased risk/benefit from testing
   - Adequately incorporated into my practice
   - Not adequately incorporated into my practice
   - Not part of my practice
   - Need more information about this activity/can’t assess
3. Please describe barriers related to incorporating any of the above activities you consider important that are not part of your practice. _________________________

The following questions pertain to Genetic Testing – Discussion and Strategy.

1. Explain the genetic testing process
   - Adequately incorporated into my practice
   - Not adequately incorporated into my practice
   - Not part of my practice
   - Need more information about this activity/can’t assess
2. Discuss rationale for the best person to test
   - Adequately incorporated into my practice
   - Not adequately incorporated into my practice
   - Not part of my practice
   - Need more information about this activity/can’t assess
3. Describe test methods (e.g., techniques, research vs. clinical testing)
   - Adequately incorporated into my practice
   - Not adequately incorporated into my practice
   - Not part of my practice
   - Need more information about this activity/can’t assess
4. Explain cost/turn-around time/insurance coverage issues
   - Adequately incorporated into my practice
   - Not adequately incorporated into my practice
   - Not part of my practice
   - Need more information about this activity/can’t assess
5. Address psychological, ethical implications
   - Adequately incorporated into my practice
   - Not adequately incorporated into my practice
   - Not part of my practice
   - Need more information about this activity/can’t assess
6. Address the risks, benefits, and limitations of genetic testing
   - Adequately incorporated into my practice
   - Not adequately incorporated into my practice
   - Not part of my practice
   - Need more information about this activity/can’t assess
7. Describe potential outcomes (positive, true negative, vs. uninformative)
   - Adequately incorporated into my practice
   - Not adequately incorporated into my practice
   - Not part of my practice
   - Need more information about this activity/can’t assess
8. Discuss importance of sharing information with at-risk family members
   - Adequately incorporated into my practice
   - Not adequately incorporated into my practice
   - Not part of my practice
   - Need more information about this activity/can’t assess
9. Please describe barriers related to incorporating any of the above activities you consider important that are not part of your practice. _________________________

The following questions pertain to Informed Consent (for clinical and/or research testing)

1. Protect autonomy/privacy/confidentiality
   - Adequately incorporated into my practice
   - Not adequately incorporated into my practice
   - Not part of my practice
   - Need more information about this activity/can’t assess
2. Discuss alternatives to testing (e.g., empiric risk management)
   - Adequately incorporated into my practice
   - Not adequately incorporated into my practice
   - Not part of my practice
   - Need more information about this activity/can’t assess
3. Please describe barriers related to incorporating any of the above activities you consider important that are not part of your practice. _________________________
